# Indian Journal of Human Genetics in PubMed: Cautious but confident steps

**DOI:** 10.4103/0971-6866.64935

**Published:** 2010

**Authors:** Kanjaksha Ghosh

**Affiliations:** National Institute of Immunohaematology, K.E.M Hospital Campus, Parel, Mumbai, India

The Indian Journal of Human Genetics is now in the sixteenth year of publication. This journal is the mouth piece of the Indian Society of Human Genetics, which was established way back in 1974, with a few scientists from Mumbai, Pune, Kolkata, Delhi, and Hyderabad, under the leadership of Dr. L D Sanghvi. It is clearly seen that the founder members were initially not in favor of starting their own journal at a time when the organization had just taken shape and was growing. At that time the main emphasis of the founder members was to make the subject of human genetics attractive and interesting.

The society had to wait 21 years before the indefatigable Prof. J.S. Murthy, from Hyderabad, started the journal, and its continuity became uncertain following the sudden and unexpected demise of Prof. Murthy, but Prof. Dipika Mohanty, former Director of the Institute of Immunohematology (Presently renamed National Institute of Immunohematology), was bold enough to take up the responsibility as the editor for the journal. Hence, in the year 2000, only one issue of the journal was published. The financial state of the journal was also not sound and for understandable reasons the authors were reluctant to publish their articles in the journal. For the last 10 years this journal is being published from the National Institute of Immunohematology. I took over as the editor of the journal in 2005, after Prof. Dipika Mohanty superannuated. Since that time, with the continuous efforts of my Associate editors, Dr. Malay Mukherjee, and now Dr. Ajit Gorakshakar, it has been possible to improve the quality and representibility of the journal and its articles over the years.

In place of two issues per year till 2004, we started bringing out three issues per year from 2005. The preliminary efforts of the publisher of the journal, that is, Medknow Publishers, and Media Pvt. Ltd. is worth recording here. Without the help of the CEO of this agency, Dr. D. K. Sahu, this journal could never have reached the position it has reached now. Through his continued efforts and the efforts of all my scientists at the Institute we could raise funds to run the journal and could index the journal with a host of indexing agencies, and we are expecting to get its impact factor known by the end of 2010.

This year your society’s only journal touched a new milestone by getting indexed in PubMed. Hence, today this journal is indexed with all the important agencies of the world. In addition to the individuals I have already mentioned earlier in the text, I have to mention the names of all the authors across the globe, including late Dr. Ernest Beutler, who helped us by submitting articles to this relatively unknown journal at that time. They did so with an implicit faith that in a country of one billion people, having thriving scientific manpower, a journal on human genetics must be present, at least to learn the important genetic lessons that mother nature teaches every day, through 30 million births in a year in the country, with a reasonably strict code of endogamy and high rates of consanguinity.

Today their faith in this journal is vindicated. Sixteen years is not a long time to assess a journal’s worth. However 16 years in also ample time when we should have been able to take the journal to a greater height. However, let us not miss the opportunity now. With more than 1000 members in the Indian Society of Human Genetics and with the society’s journal indexed in ‘PubMed’ there should be no hesitation by learned members to put their best work in the journal, and work for the journal, so that it becomes more visible internationally. Young members of the society should contribute by submitting interesting observations made by them. They should publish good testable hypothesis in this journal and also should raise controversies and publish constructive criticisms on published articles as well as in the in the cutting edge areas of the science of genetics and genomics through correspondence and through original articles and perspectives.

I request all the members of the society to visit the journal website at www.ijhg.com. It will be a pleasure to see how well Medknow Publishers have created the website. Each article has its individual statistics of utility. You can see how often the articles in the journal have been visited, how often the article PDF has been downloaded or the article is cited. In addition you can also map the authors’ and the reviewers’ institutions across the world. The impact factor and citations are two important indices of any journal and an individual article’s utility, but I am sure that in future, the number of times an article has been visited or PDF downloaded will also be used by scientometrists to understand the interest in a particular article, by readers who do not always publish articles. Getting indexed in PubMed is one of the landmarks in the short life of this journal. I am sure with all your help this journal will be able to immortalize itself.

